# Surgical removal and controlled trypsinization of the outer annulus fibrosus improves the bioactivity of the nucleus pulposus in a disc bioreactor culture

**DOI:** 10.1186/s12891-016-0990-2

**Published:** 2016-03-22

**Authors:** Pei Li, Rongmao Shi, Daosen Chen, Yibo Gan, Yuan Xu, Lei Song, Songtao Li, Qiang Zhou

**Affiliations:** Department of Orthopedic Surgery, Southwest Hospital, Third Military Medical University, Gao Tan Yan 29, Chongqing, 400038 China; Department of Orthopedic Surgery, Kunming General Hospital of Chengdu Command, Kunming, Yunnan 650032 China; The 91245 Troops of the Chinese People’s Liberation Army, Huludao, Liaoning 125000 China; Department of Orthopedic Surgery, Xinqiao Hospital, Third Military Medical University, Chongqing, 400038 China; Department of Orthopedic Surgery, No. 181 hospital of PLA, Guilin, Guangxi 541002 China

**Keywords:** Intervertebral disc, Degeneration, Organ culture, Nucleus pulposus

## Abstract

**Background:**

The maintenance of nucleus pulposus (NP) viability in vitro is difficult. The annulus fibrosus (AF) pathway reflects one nutrient transport channel and may have an important effect on NP viability in disc organ cultures. The present study describes a feasible disc pre-treatment involving the AF and investigates its efficacy in improving NP bioactivity in an in vitro disc bioreactor culture.

**Methods:**

Rabbit discs that were randomly assigned to the experimental group (EG) were pretreated via the surgical removal and controlled trypsinization of the outer AF. The discs in the control group (CG) did not receive any special treatment. All discs were organ-cultured in a self-developed bioreactor. Solute transport into the central NP was measured using a methylene blue solution. On days 7 and 14, histological properties, cell viability, cell membrane damage, gene expression and matrix composition within the NP in these two groups were compared with each other and with the corresponding parameters of fresh NP samples. Additionally, the structures of the outer AF and the cartilage endplate (CEP) following pre-treatment were also assessed.

**Results:**

The outer AF in the EG became disorganized, but no specific changes occurred in the CEP or the inner AF following pre-treatment. The discs in the EG exhibited increased penetration of methylene blue into the central NP. On days 7 and 14, the NP bioactivity in the EG was improved compared with that of the CG in terms of cell viability, cell membrane damage, gene expression profile and matrix synthesis. Moreover, cell viability and matrix synthesis parameters in the EG were more similar to those of fresh samples than they were to the same parameters in the CG on day 14.

**Conclusions:**

Using this disc pre-treatment, i.e., the surgical removal and controlled trypsinization of the outer AF, NP bioactivity was better maintained for up to 14 days in an in vitro disc bioreactor culture.

## Background

Intervertebral disc degeneration (IDD) with or without low back pain (LBP) is a worldwide disease [1]. Current treatments, including surgery and conservative therapy, aim to alleviate the severity of the pain to a certain extent but do not address the onset of IDD [2, 3]. Current treatments including regenerative tissue engineering and biological therapy still require additional research [4–6].

The intact disc organ culture system is a suitable platform for studying IDD due to the ability to control biochemical and biomechanical boundary conditions and the retention of the native extracellular matrix (ECM) [7]. However, due to the avascular nature of the intervertebral disc (IVD), nutrient supply to the central disc is insufficient; thus, the central nucleus pulposus (NP) has a low density of the viable cells that are responsible for synthesizing the ECM [8]. Dynamic compression is helpful for enhancing the nutrient supply to the central NP through convective transport. However, some disc studies, such as drug-effectiveness tests and gene therapy-related studies, do not require the involvement of dynamic compression. Moreover, mechanical load application devices are expensive and can be complex to operate. Hence, an alternative method that can maintain NP viability in an in vitro disc culture is necessary for studies that do not require the involvement of a mechanical load.

Under physiological conditions, nutrients diffuse in and out of the central disc through the cartilage endplate (CEP) and the annulus fibrosus (AF) [9]. Because the CEP contains many nutrient channels that are permeable to many substances (e.g., glucose, oxygen and amino-acids) [10], several studies have focused on the CEP in efforts to enhance nutrient diffusion into the central NP [11, 12]. The AF is a vascular tissue at birth, but the blood vessels of the inner annulus gradually disappear with age until only the outer AF retains a few blood vessels [13]. Previous studies suggest that although the AF is not a major nutrient diffusion pathway, it can affect the nutrient supply to the disc cells and can even become the primary nutrient transport pathway when nutrient diffusion via the CEP route is impaired [14, 15]. To our knowledge, no previous studies have attempted to increase nutrient diffusion into the central disc through the AF pathway.

It is well known that without any convective transport induced by external mechanical compression, nutrients move into the central NP primarily via diffusion, and the efficiency of this movement is inversely proportional to the nutrient diffusion distance [16]. Moreover, the distance from the peripheral AF to the NP has been estimated to be much greater than that from the CEP to the NP [17]. Finally, the compact characteristic of the outer AF may hinder nutrient diffusion into the central NP to some extent. Therefore, shortening the nutrient diffusion distance of the AF pathway and loosening the superficial AF could theoretically improve the nutrient supply to the central NP and thereby enhance NP viability in disc explant cultures. Here, we present a pre-treatment that involves the surgical removal and controlled trypsinization of the outer AF prior to the initiation of disc bioreactor culturing. We tested the hypothesis that this pre-treatment would improve NP bioactivity in disc bioreactor cultures. To examine this hypothesis, NP samples were analyzed for histological characteristics, cell viability, biochemical content, gene expression and matrix protein expression.

## Methods

### Intervertebral disc harvesting

Twenty-four healthy New Zealand white rabbits (3–4 months old, male and female) were used in this study. All animal experiments were approved by the Ethics Committee of Southwest Hospital affiliated with the Third Military Medical University [SYXK (YU) 2012-0012]. Briefly, 5 min before sacrificing the rabbits via air embolism, 5,000 IU of heparin sodium was injected via an ear vein to prevent blood clotting in the endplate capillaries. After the spinal column was harvested under sterile conditions, as much of the vertebral bone tissue as possible was removed from the isolated thoracolumbar and lumbar discs (T11-L5) with a scalpel blade to obtain discs with the CEPs. Due to the differences between the different vertebral levels, discs from the same levels were used for the same assays in this study. For example, the same 3 discs (Th11/12, Th12/L1 and L1/2) from different animals were used for the GAG content analysis. Consequently, discs from the same levels used for the same assay were similar in size and were anatomically adjacent, which was helpful for avoiding biases that might result from variations in disc size and other factors.

### Intervertebral disc pre-treatment and bioreactor cultures

**Fig. 1:**
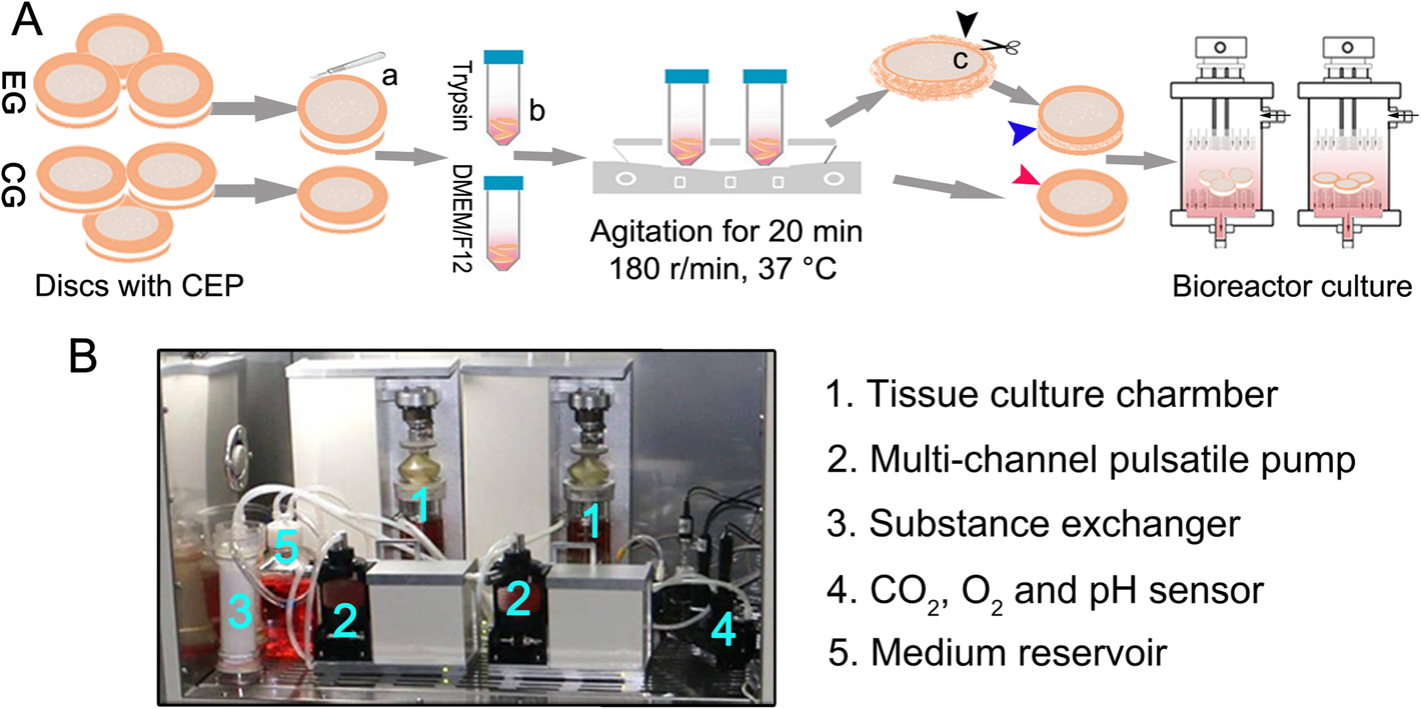
Illustration of the intervertebral disc pre-treatment and the bioreactor system used for the rabbit disc cultures. **a** Rabbit discs with cartilage endplates were isolated and assigned to the experimental group (EG) or control group (CG). First, approximately the outermost one-third of the annulus fibrosus (AF) of the discs in the EG, but not in the CG, was removed (*a*). Then, the discs in the EG were placed in a 0.25 % trypsin solution to loosen the network of superficial AF, while the discs in the CG were placed in DMEM/F12 medium (*b*). After trypsinization, the digested superficial AF tissue in the EG was cut out (*c*). Finally, all discs were cultured in the chambers of a self-developed bioreactor system. **b** Overview of the bioreactor system, which consists of the five labeled functional units

Isolated discs were treated with our disc pre-treatment protocol [experimental group (EG)] or were untreated [control group (CG)]. Specifically, the outer one-third (approximately) of the AF was removed in the EG with a No. 11 scalpel blade under a dissecting microscope, and the AF was not removed in the CG. Thereafter the discs in the EG were placed in a 0.025 % trypsin solution supplemented with 1 % (v/v) penicillin-streptomycin (HyClone) and 20 mM sodium citrate (Sigma) to loosen the network of the superficial AF. The discs in the CG were placed in DMEM/F12 medium (HyClone) supplemented with 1 % (v/v) penicillin-streptomycin and 20 mM sodium citrate. After agitation (37 °C, 180 r/min) for 20 min and the removal of the digested superficial AF tissue in the EG with ophthalmic scissors, discs were transferred to the tissue culture chambers of a self-developed bioreactor (Fig. 1). DMEM/F12 medium supplemented with 10 % fetal bovine serum (Gibco), 1 % penicillin/streptomycin and 0.025 mg/mL ascorbic acid (Sigma) was circulated at a rate of 10 mL/min. All discs were cultured for 14 days under standard conditions (37 °C, 21 % O_2_, 5 % CO_2_, and 100 % humidity) and the media were replaced every 3 days.

### Methylene blue transport into the NP tissue

Methylene blue (373.90 Dalton, Jumpcan Pharmaceutical Group, China) is widely used in clinical practice due to its high diffusion efficacy in human tissues. To indirectly determine if this disc pre-treatment improves solute transport into the central NP, discs in the EG and CG were incubated with a 3-mL methylene blue injection. After 16 h, one side of the CEP was isolated. Then, the methylene blue staining of the NP tissue was recorded using a digital camera, and the staining intensity within the central NP region was quantified using Image-Pro Plus software (Version 5.1).

### Histological analysis

Discs were sequentially fixed with 4 % paraformaldehyde, decalcified with 10 % ethylenediaminetetraacetic acid and embedded in paraffin for sectioning. Hematoxylin and eosin (HE) staining and toluidine blue staining were performed on cross-sectional slices (5 μm thick) to evaluate cell morphology and proteoglycan (PG) distribution, respectively, within the NP. Staining intensity of toluidine blue was quantified using Image-Pro Plus software (Version 5.1). In addition, we also performed HE staining on the AF (the outer and the inner regions) and the CEP (the cross section and the sagittal section) to observe changes within the AF and CEP after the pre-treatment.

### Detection of disc cell damage

To evaluate membrane damage in the disc cells in these two groups during the culture period, a lactic dehydrogenase (LDH) release assay was performed. Briefly, discs were incubated in 6-well plates under standard conditions, and the media were replaced every other day. One milliliter of culture medium was collected at each medium exchange to determine LDH activity on days 2, 4, 6, 8, 10, 12 and 14 with a quantification kit (Nanjing Jiancheng, China).

### Cell viability evaluation

Cell viability was detected using nitrotetrazolium blue chloride (NBT, Beyotime, China), which stains live cells, and 2-(4-amidinophenyl)-6-indolecarbamidine dihydrochloride (DAPI, Beyotime, China), which stains cell nuclei, according to a previously reported method [18]. NP cell viability was calculated based on a viability percentage [Live Cells/Total Cells].

### Measurement of the biochemical contents

At each sampling point, NP samples were isolated to measure the glycosaminoglycan (GAG) and hydroxyproline (HYP) contents. One group of NP samples was lyophilized for 24 h and weighed to determine the dry weight. Then, the dried NP samples were digested at 60 °C for 24 h in 1 mL of water containing 5 mg/mL papain,0.2 mol/L NaCl, 0.01 mol/L cysteine hydrochloride, 0.1 mol/L CH_3_COONa and 0.05 mol/L Na_2_-EDTA (Sangon, Biotech Co., Ltd., China). Next, the GAG content was calculated using a dimethyl methylene blue (DMMB) assay [19] in which chondroitin sulfate from shark cartilage was used as a standard. Another group of NP samples was weighed to determine their wet weights, and the HYP content was then determined using an HYP quantification kit (NanJing JianCheng, China) according to the manufacturer’s instructions.

### Quantification of gene expression

The relative expression of relevant genes (i.e., aggrecan, collagen II, collagen I, ADAMTs-4, MMP-3, TIMP-1 and TIMP-3) was assessed via real-time PCR as previously described [20]. The primers of the target genes (Table 1) were designed using Primer 5.0 software. The highly conserved GAPDH gene was used as the reference gene, and the expressions of the target genes were quantified based on the 2^―△△Ct^ method.

**Table 1:** Primers of target genes

Gene	Accession number	Forward (5′-3′)	Reverse (5′-3′)
GAPDH	NM_001082253.1	GACCACTTTGTGAAGCTCATTTC	GTGGTTTGAGGGCTCTTACTC
Aggrecan	XM_002723376.1	CTCCCTGGTAGATACTCCATTG	CTGGAGGGAAGTCCAGATATT
Collagen II	NM_001195671.1	AGCGGTGACTACTGGATAGA	CTGCTCCACCAGTTCTTCTT
Collagen I	XM_002719108.1	GGTACAGTGAAGGCGAAATATG	ACAGTCCTTGGTGTCTTCA
ADAMTs-4	XM_002715171.1	AGCGCCCACTTCATCACCAAC	GGGCGAGTGCTTGGTCTGG
MMP-3	NM_001082280.1	GTTCCTGATGTTGGTCACTTC	GCAGATCCGGTGTGTAATTC
TIMP-1	NM_001082232.2	TACTCCCACAAATCCCAGAA	AACCACGAAACTGCAAGTC
TIMP-3	NM_001195682.1	CGTGTTTATGATGGCAAGGT	AGGTGGTAGCGATAGTTCAG

### Immunohistochemistry

To analyze protein expression of collagen II and aggrecan within the NP, we performed immunohistochemical staining as previously described [20]. Primary antibodies against aggrecan (Novus, NB120-11570) and collagen II (Abcam, ab34712) were used at a dilution of 1:200. The primary antibodies were replaced with bovine serum albumin in the negative controls. All sections were viewed under a light microscope (Olympus BX51).

### Western blotting analysis

To explore matrix protein metabolism within the NP, we examined the protein expression of collagen II and ARGxx (an indicator of aggrecan degradation products) as previously described [21]. Primary antibodies against ARGxx, collagen II and GAPDH (ab3773, ab34712 and ab8245, respectively) were used at a dilution of 1:1000. Protein expression was quantified with ImageJ software and normalized to GAPDH expression.

### Statistical analysis

All data are presented as the mean ± SD and were analyzed using SPSS software (Version 13.0). Each experiment in this study was performed in triplicate. After testing for homogeneity of variance, the significant differences between the two groups (i.e., CG and EG at each time point) were assessed using independent-sample T tests, whereas the significant differences between the fresh group, CG at day 14 and EG at day 14 were analyzed via a one-way analysis of variance (ANOVA) followed by post hoc LSD tests. Significant difference was defined based on a *p*-value < 0.05.

## Results

### Methylene blue transport

**Fig. 2:**
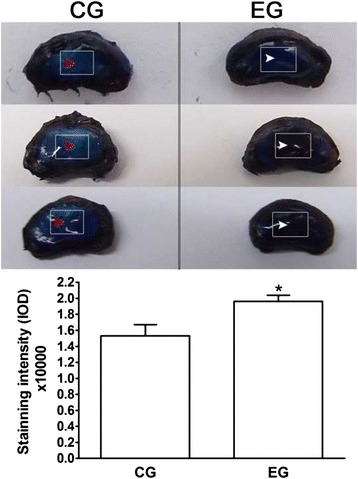
Exposure of the nucleus pulposus (NP) after methylene *blue* incubation. Arrows (*white* and *red*) indicate that methylene *blue* diffused into and stained the NP tissue and indicate a higher concentration of methylene *blue* within the central NP in the EG. The histogram shows the quantified results of the staining intensity in the CG and EG. CG: control group; EG: experimental group. Data are expressed as the mean ± SD (*n* = 3). *: *p* < 0.05 vs. the CG

Representative images indicate that methylene blue could diffuse into the central NP and that the penetration of methylene blue into the NP tissue in the EG exceeded that in the CG (Fig. 2).

### Histology

**Fig. 3:**
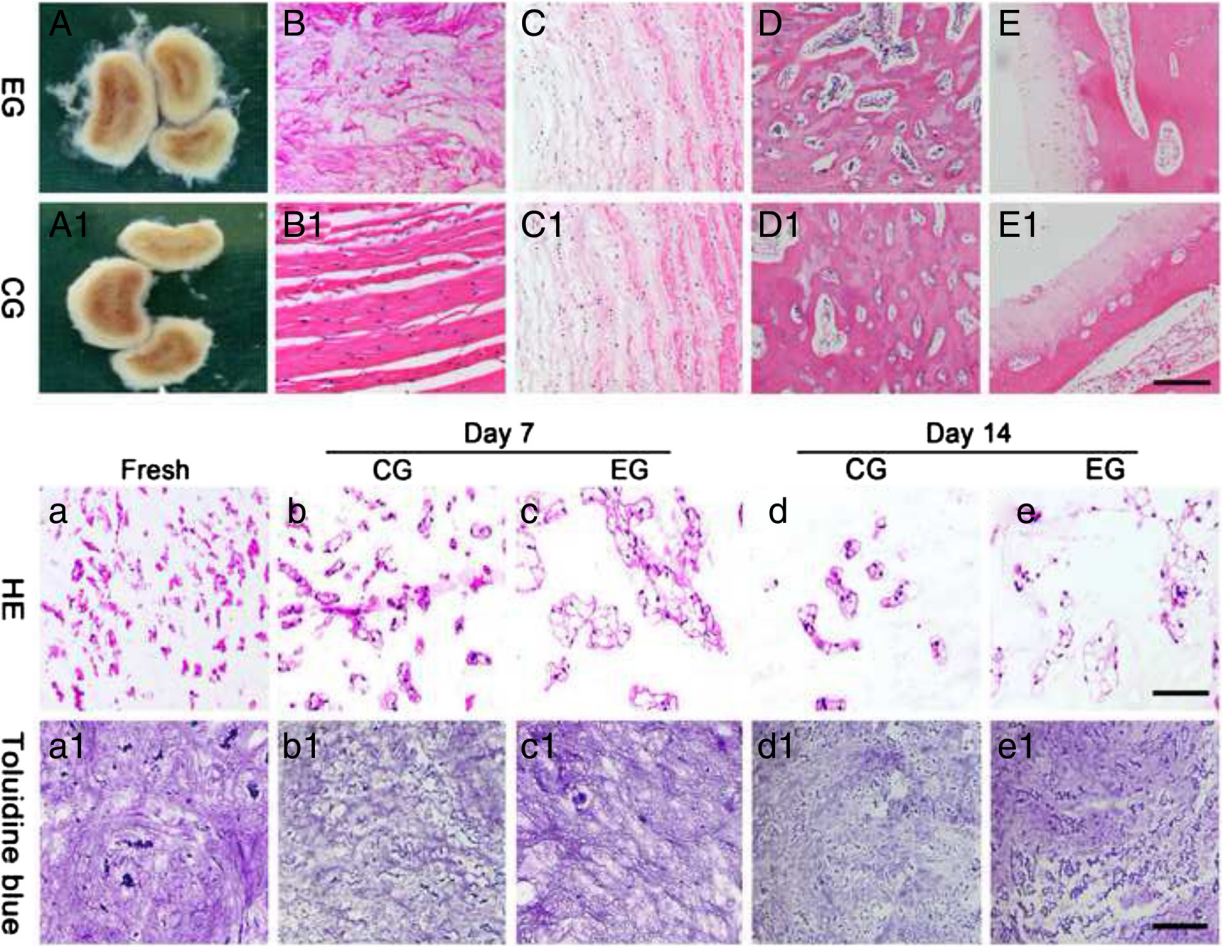
Histological observation of pretreated discs and cultured discs. The upper half depicts the gross disc appearance and the HE staining of the annulus fibrosus (AF) and the cartilage endplate (CEP) in the EG and CG after pre-treatment. B, B1: the superficial AF; C, C1: the inner AF. D, D1: cross section; E, E1: sagittal section. The lower photomicrographs show the analyses of HE staining (b-e) and toluidine blue staining (b1-e1) in the nucleus pulposus (NP) tissue of the EG and CG at days 7 and 14. Fresh samples (a, a1) were also used for comparison. CG: control group; EG: experimental group. Magnification: B-E, B1-E1 and a-e: 200x, scale represents 100 μm (*n* = 3); a1-e1: 100x, scale represents 200 μm (*n* = 3)

After the discs were pretreated prior to bioreactor culturing, both the general and microscopic appearances revealed that the superficial AF in the EG but not in the CG became disorganized, and no obvious differences in the CEP between the CG and EG were found (Fig. 3A-E, A1-E1). After 7 and 14 days of culture, NP cells displayed a large and rounded morphology, and no apparent abnormalities in these two groups relative to the appearance of the NPs from the fresh group were identified (Fig. 3a-e). However, toluidine blue staining indicated that the PG content in the CG was significantly decreased at both 7 and 14 days compared with that of the EG or fresh NP group (Fig. 3 a1-e1).

### Cell membrane damage

**Fig. 4:**
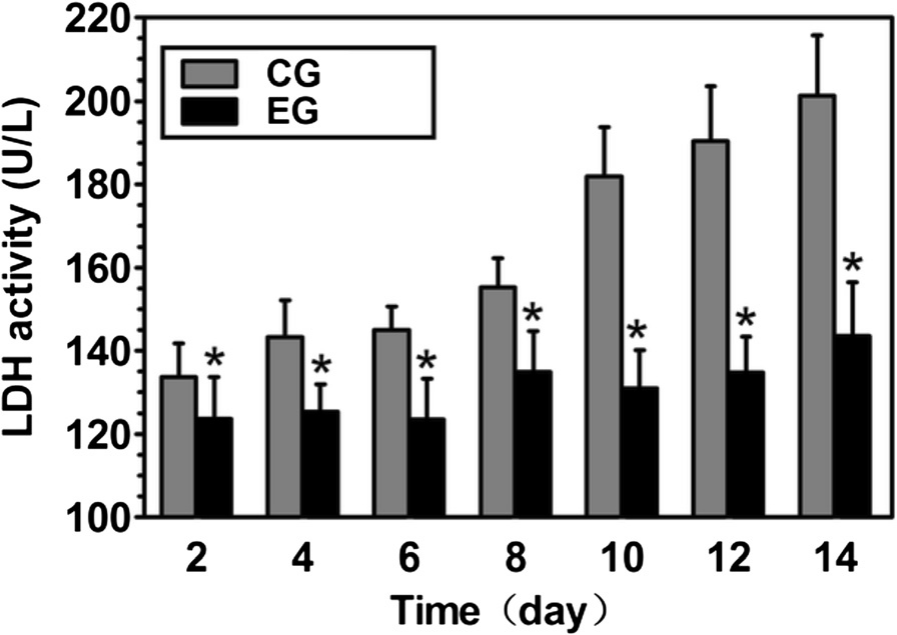
Analysis of lactate dehydrogenase (LDH) activity on days 2, 4, 6, 8, 10, 12 and 14. CG: control group; EG: experimental group. Data are expressed as the mean ± SD (*n* = 3). *: *p* < 0.05, vs. the CG

Over the entire culture duration, LDH activity in the CG dramatically increased, whereas this activity remained stable in the EG. Moreover, the LDH activity in the CG was significantly increased compared with that in the CG on days 2, 4, 6, 8, 10, 12 and 14 (all *p*-values < 0.05, Fig. 4).

### Cell viability

**Fig. 5:**
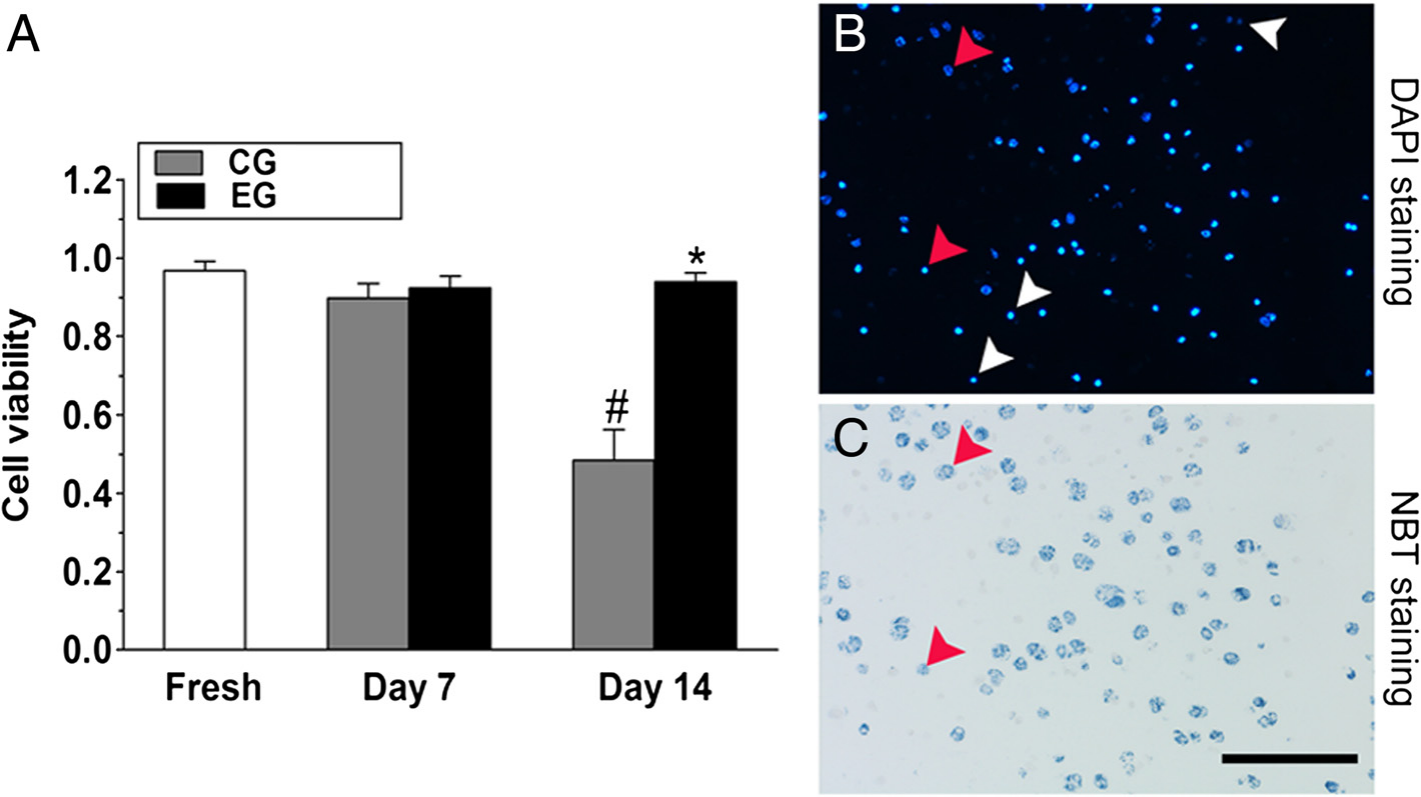
Nucleus pulposus (NP) cell viability analysis. **a: ** Comparison of NP cell viability between fresh discs and discs from the EG and CG. Data are expressed as the mean ± SD (*n* = 4). *: *p* < 0.05 vs. the CG; #: *p* < 0.05 vs. fresh discs. **b** and **c: ** Live and dead cells were visualized using fluorescence microscopy. Viable cells were double stained with DAPI and NBT (red arrow) while dead cells were only stained with DAPI (white arrow). CG: control group; EG: experimental group. Magnification: 200x, scale represents 100 μm

NBT and DAPI staining revealed that the live NP cells were simultaneously stained dark blue (NBT) and bright fluorescent blue (DAPI), whereas the dead NP cells only showed nuclear staining (Fig. 5b, c). At day 7, NP cell viability did not differ between the CG and EG (*p* = 0.347). At day 14, although no remarkable differences in the NP cell viability of fresh discs and discs in the EG were found, the NP cell viability in the CG was seriously compromised compared with that of the EG and fresh discs (*p* < 0.05, Fig. 5a).

### Biochemical content

**Fig. 6:**
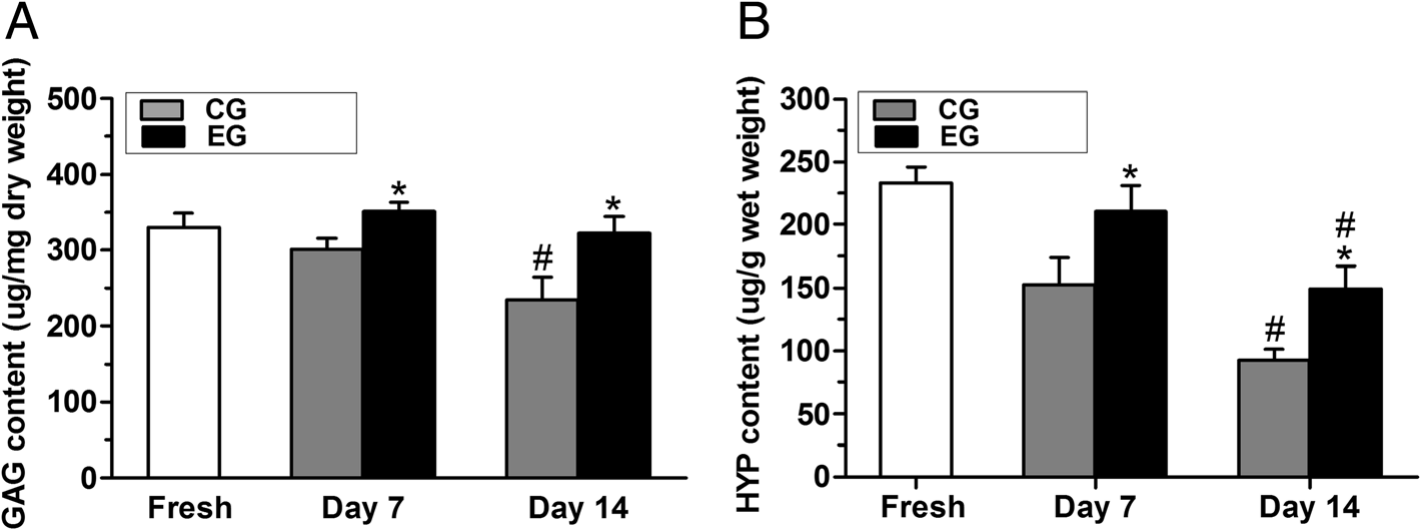
Quantification of the biochemical content within the nucleus pulposus (NP). **a**: glycosaminoglycan (GAG) content. **b**: hydroxyproline (HYP) content. CG: control group; EG: experimental group. Data are expressed as the mean ± SD (*n* = 3). *: *p* < 0.05 vs. the CG; #: *p* < 0.05 vs. fresh discs

On days 7 and 14, the GAG content in the CG was significantly decreased compared with that of the EG (*p* < 0.05). When compared to the fresh samples on day 14, the GAG content in the CG was decreased (*p* < 0.05). Similarly, the HYP content in the CG was also decreased compared with that in the EG (*p* < 0.05). At day 14, in both the CG or EG, the HYP content was significantly decreased compared with that in the fresh samples (*p* < 0.05, Fig. 6).

### Gene expression

**Fig. 7:**
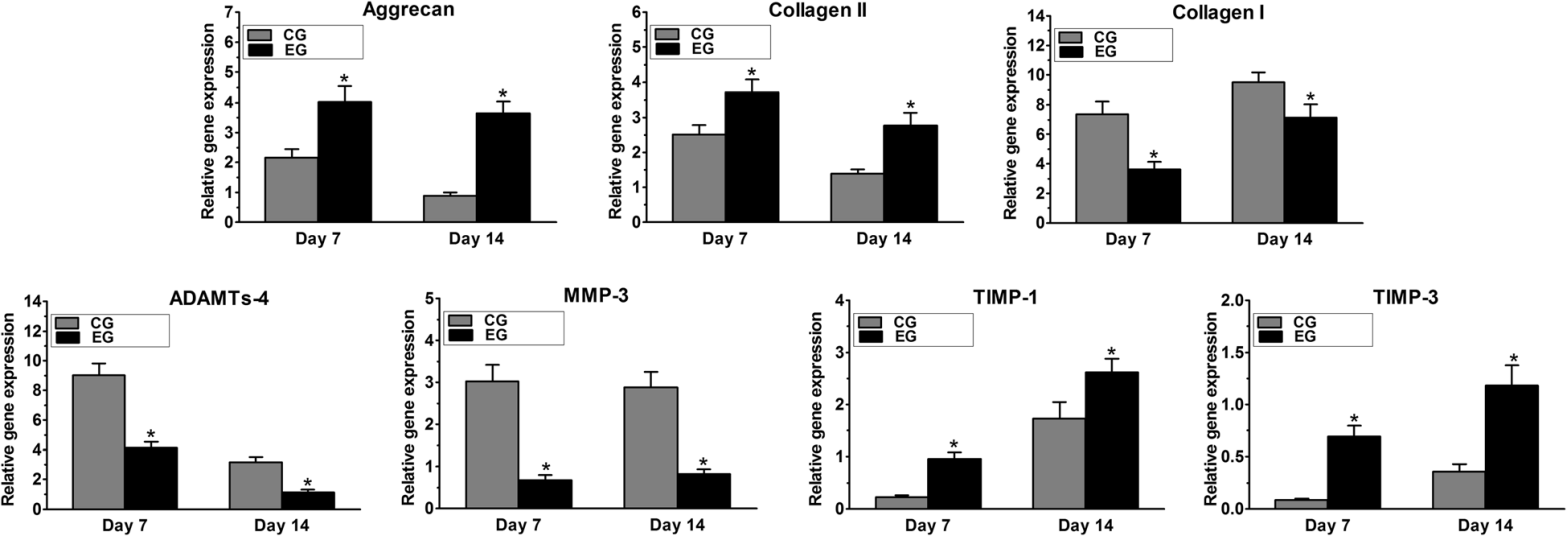
Gene expression in nucleus pulposus (NP) cells of the cultured discs. CG: control group; EG: experimental group. Data are expressed as the mean ± SD (*n* = 3). *: *p* < 0.05 vs. the CG

In the EG, aggrecan and collagen II were significantly up-regulated compared with expression in the CG, whereas collagen I expression was down-regulated (*p* < 0.05). The expression of anabolic metabolism-related enzymes (i.e., TIMP-1 and TIMP-3) was also elevated in the EG compared with their expression in the CG (*p* < 0.05). In contrast, the expression of matrix-degradation enzymes (i.e., ADAMTs-4 and MMP-3) was up-regulated in the CG compared with their expression in the EG (*p* < 0.05, Fig. 7).

### Matrix protein expression

**Fig. 8:**
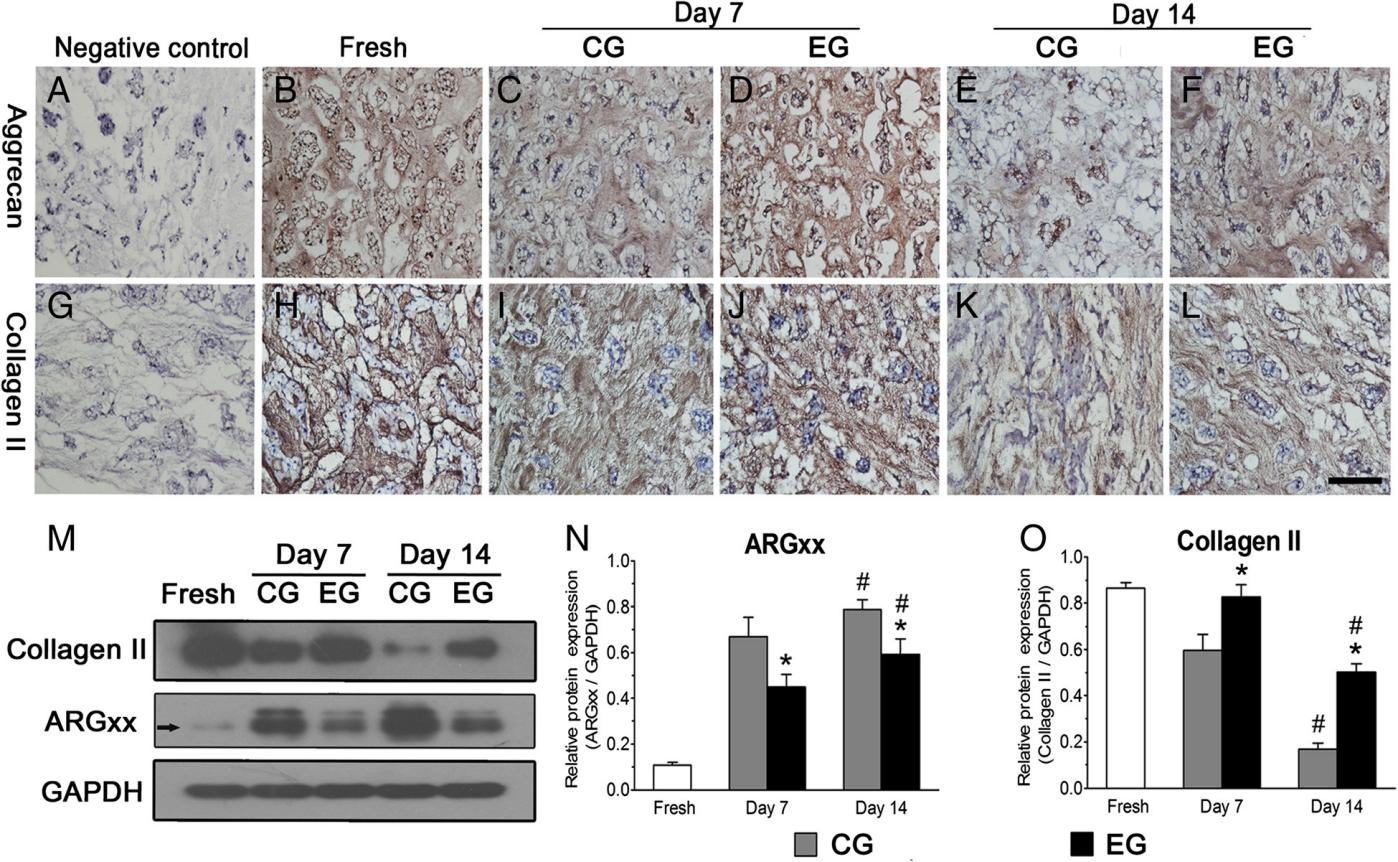
Immunohistochemistry and western blot analysis of protein expression. Photomicrographs **a**-**l** show immunohistochemical staining for collagen II and aggrecan within the nucleus pulposus (NP) in the EG and CG at days 7 and 14. **b**, **h**: fresh NP. **a**, **g**: negative controls. Magnification: 200x, scale represents 100 μm (*n* = 3). The results (**m**-**o**) of the western blot analysis of aggrecan degradation products (ARGxx) and collagen II within the NP in the EG and CG at days 7 and 14. Fresh samples were also used for comparison. CG: control group; EG: experimental group. Data are expressed as the mean ± SD, *n* = 3. *: *p* < 0.05 vs. the CG

Immunohistochemistry revealed that the staining intensity for aggrecan and collagen II in the EG was increased compared with that in the CG on days 7 and 14 and that the staining intensity for aggrecan and collagen II in the CG was significantly decreased compared with the that in the fresh samples on day 14 (Fig. 8a-l). Similarly, western blot analysis showed an increased expression of collagen II in the EG compared with its expression in the CG on days 7 and 14 and a decreased expression of collagen II in both groups compared with its expression in the fresh samples on day 14 (*p* < 0.05, Fig. 8m, o). Additionally, the expression of ARGxx was decreased in the EG compared with its expression in the CG on days 7 and 14, whereas it was increased in both groups on day 14 compared with its expression in the fresh samples (*p* < 0.05, Fig. 8m, n).

## Discussion

The present study introduced a disc pre-treatment prior to bioreactor culturing and evaluated its efficacy in improving NP bioactivity in disc explant cultures. This disc pre-treatment, described as the surgical removal and controlled trypsinization of the outer one-third of the AF, supports our hypothesis and provides a practical method for enhancing NP viability in disc bioreactor cultures. This disc pre-treatment resulted in an improvement in NP bioactivity over 14 days of culture. Compared with other in vitro disc culturing systems, this disc perfusion culture resulted in an improved NP bioactivity that was comparable to those that have previously been achieved with certain mechanical stimuli. However, we also realize that these results come at the expense of tissue integrity and the mechanical properties of the disc as a whole. Therefore, we suggest that this system, which involves no mechanical stimulation, is appropriate for studies on the effects of certain biochemical stimuli on NP bioactivity in vitro. Such studies will be helpful for improving our understanding of non-mechanically related degenerative changes within the NP.

In this disc pre-treatment, we trypsinized the surface of the remaining AF to reduce its density after the outermost one-third of the AF was surgically removed. After several initial trials of various durations (e.g., 10 min, 20 min, 30 min, 1 h and 1.5 h), 20 min was chosen as a suitable digestion period because it resulted in no grievous harm to the adjacent CEP or to the inner AF (Fig. 3). Methylene blue was used to test solute transport into the central NP in the present study. The higher concentration of methylene blue in the central NP in the EG suggests that our disc pre-treatment resulted in enhanced solute transport into the central NP.

Successful IVD culturing requires a sufficient nutrient supply to preserve disc cell viability. In the present study, a high level of NP cell viability, which was comparable to that of the fresh NP and higher than that of the CG, was observed in the EG and was maintained for at least 14 days. Similarly, the cell membranes were better protected in the EG than in the CG. Together, these findings indicate that NP cell viability was better maintained in the EG than in the CG. Previously, methods that enhance nutrient transport into the central NP through the CEP pathway, such as pre-mortem heparinization and post-mortem removal of vertebral bone, have also been reported to improve NP viability in vitro [12, 21]. Consistently, considerable cell viability was also observed in the CG on day 7. However, this viability was significantly decreased compared with that of the EG on day 14. In previous studies that employed disc pre-treatments similar to the one applied to the CG, high levels of NP cell viability over periods exceeding 7 days have been reported [22, 23]. One possible contributor to this is the application of mechanical stimulation, which might induce convective transport and/or activate mechanotransduction pathways to preserve NP cell viability. Hence, it may not be appropriate to simply compare the cell viability between different studies without considering the different methodologies used.

In the EG, gene expression analysis showed an up-regulations of aggrecan, collagen II, TIMP-1 and TIMP-3 and a down-regulations of MMP-13 and ADAMTS-4. These findings suggest that anabolism was stimulated in the NP cells. In contrast, a catabolic gene expression profile [24] was observed in the CG; specifically, the down-regulation of aggrecan and collagen II and the up-regulation of collagen I and degenerative enzymes were observed. This phenomenon was also observed in another study that demonstrated a similar pattern of aggrecan, collagen II and collagen I gene expression [23]. Adequate nutrient supply to the central NP has important effects on NP cell behavior. The pronounced effect of nutrient supply on NP cell metabolism has been investigated by Rinkler et al. [25], who reported that aggrecan and collagen II tend to be down-regulated, whereas matrix metalloproteinases (MMPs) tend to be up-regulated during periods of glucose deprivation. Based on these findings, we speculate that the gene expression profile observed in the CG was partially attributable to insufficient nutrient supply, which induced degenerative changes, and the anabolic gene expression profile of the EG was probably due to the enhanced nutrient supply caused by the disc pre-treatment.

The turnover of ECM components is another critical parameter for validating the utility of this disc pre-treatment. In our study, ECM production within the NP in the EG was increased compared with that in the CG on days 7 and 14, whereas the opposite pattern was observed for ARGxx expression in these two groups (Figs. 6 and 8). These findings suggest that the matrix-synthesizing ability of NP cells in the EG was enhanced. Furthermore, the GAG content and aggrecan protein expression in the EG remained similar to those of the fresh samples on day 14. These findings are not consistent with the results of Lee et al. [26] or Haschtmann et al. [23], who demonstrated decreased PG contents within the NP in their disc culture systems. This discrepancy may have been caused by our disc pre-treatment or by the use of a perfusion culture system that is able to increase nutrient exchange efficacy and remove metabolic byproducts in a timely manner. Additionally, either the HYP content or the collagen II protein expression in the EG was still decreased compared with the content of the fresh samples. This unfavorable result may have been due to the absence of certain biological or physicochemical factors that influence collagen protein metabolism in vitro [27]. As Heather et al. [28] discussed, a level of nutrient supply that is sufficient for maintaining cell viability may not be enough to maintain matrix synthesis. Hence, the relatively insufficient nutrient supply possibly contributed to the decreased matrix synthesis in the CG.

The disc explant culture system established via this disc pre-treatment possesses several limitations. First, Smith et al. demonstrated that the AF is necessary for maintaining the overall mechanical properties of the disc [29, 30]. Surgical removal of the outer AF weakens the ability of the AF to restrict NP swelling, which may in turn affect disc cell biology to some extent. This new disc pre-treatment may therefore only be suitable for studies that do not involve external mechanical stimuli. Second, although the control group and the methods utilized were designed to prevent biases resulting from the vertebral level from which samples were collected and from the experimental animals, the efficiency of this disc pretreatment method for discs from larger animal should be validated, and a larger sample size is also needed to better examine the scientific questions posed in this study. Third, rabbit discs have a small geometric size, which in turn makes them poorly suited for studies involving nutrient diffusion. In the present study, although the pre-treatment was found to enhance solute transport into the central NP, the diffusion efficacy of certain nutrients (i.e., glucose and oxygen) and other relevant metabolites were not evaluated.

## Conclusion

In summary, we describe here a feasible disc pre-treatment and validate its efficacy in improving NP bioactivity in a disc bioreactor culture. This new technique, which involved the surgical removal and controlled trypsinization of the outer AF, resulted in an improved ability to maintain NP bioactivity over 14 days in terms of cell viability, gene expression and matrix synthesis. This pretreatment could be useful for the establishment of a specific stable model for studying NP biology in the presence of biochemical stimuli.

## Abbreviations

IDD: Intervertebral disc degeneration
LBP: Low back pain
ECM: Extracellular matrix
IVD: Intervertebral disc
NP: Nucleus pulposus
CEP: Cartilage endplate
AF: Annulus fibrosus
EG: Experimental group
CG: Control group
EDTA: Ethylenediaminetetraacetic acid
PG: Proteoglycan
GAG: Glycosaminoglycans
HYP: Hydroxyproline
